# Probiotics for the prevention of gestational diabetes mellitus: A meta-analysis of randomized controlled trials

**DOI:** 10.17305/bb.2024.10377

**Published:** 2024-10-01

**Authors:** Xue Li, Luwen Zhang, Yuanqi He, Dandan Zhang, Shihong Zhang

**Affiliations:** 1 Department of Obstetrics, Weihai Municipal Hospital, Cheeloo College of Medicine, Shandong University, Weihai, China; 2 Department of Obstetrics, The First Affiliated Hospital of Jinan University, Guangzhou, China; 3 Department of Gynaecology and Obstetrics, Weihai Municipal Hospital, Cheeloo College of Medicine, Shandong University, Weihai, China

**Keywords:** Gestational diabetes mellitus (GDM), probiotics, prevention, incidence, meta-analysis

## Abstract

Changes in intestinal microbiota have been shown to be involved in the development of gestational diabetes mellitus (GDM). We performed a meta-analysis to systematically evaluate the potential role of probiotics in the prevention of GDM. A systematic literature search was performed in electronic databases, including PubMed, Cochrane Library, Embase, Web of Science, Wanfang, and China National Knowledge Infrastructure (CNKI) to obtain relevant randomized controlled studies. A random-effects model was used to pool the results by incorporating the impact of the potential heterogeneity. Meta-regression and subgroup analyses were conducted to evaluate the source of heterogeneity. Fourteen studies involving 3527 pregnant women were included. Results showed that probiotics significantly reduced the incidence of GDM as compared to control (risk ratio [RR]: 0.71, 95% confidence interval [CI]: 0.52–0.96, *P* ═ 0.03) with significant heterogeneity (I^2^ ═ 73%). The meta-regression showed that the body mass index (BMI) of women was positively associated with the RR for the effect of probiotics on GDM (coefficient ═ 0.084, *P* ═ 0.01). The results of subgroup analyses also suggested that probiotics significantly reduced the risk of GDM in women with BMI < 26 kg/m^2^, but not in those with BMI ≥ 26 kg/m^2^ (*P* for subgroup difference ═ 0.001). In addition, the preventative efficacy of probiotics on GDM was remarkable in women < 30 years, but not in those ≥ 30 years (*P* for subgroup difference < 0.001). In conclusion, probiotics may be effective in reducing the risk of GDM, particularly for women with lower BMI and younger age.

## Introduction

Gestational diabetes mellitus (GDM) is a prevalent metabolic disorder that occurs during pregnancy [1, 2]. Existing literature suggests that the prevalence of GDM among pregnant individuals ranges from 15% to 20% [1]. Risk factors associated with GDM include advanced maternal age, elevated body mass index (BMI), familial history of type 2 diabetes mellitus (T2DM), and a prior history of GDM in a previous pregnancy [3]. Emerging research indicates that GDM is not only linked to immediate adverse outcomes, such as miscarriage, preterm birth, and macrosomia [4, 5], but it is also associated with a range of long-term health risks for both mothers and their offspring, including maternal and child obesity, increased risk of type 2 diabetes, and heightened maternal susceptibility to cancer and cardiovascular diseases [4, 6, 7]. Consequently, there is a pressing need for the development of innovative approaches to prevent the onset of GDM [8].

Pregnancy has been associated with disruptions in the homeostasis of intestinal microbiota, with a notable increase in actinobacteria and proteobacteria observed in 60%–70% of women [9, 10]. Studies have shown that women with GDM exhibit more pronounced alterations in gut microbiota compared to those without GDM, resembling patterns seen in non-pregnant women with T2DM [11, 12]. This suggests a potential role of gut microbiota in the development of GDM. Probiotics, as living microorganisms, play a beneficial role in restoring and maintaining the balance of gut microbiota composition [13]. In T2DM patients, the use of probiotics has been linked to a reduction in insulin resistance and enhancement of glycemic control [14, 15]. Furthermore, in females with a confirmed diagnosis of GDM, supplementation with probiotics has demonstrated improvements in hyperglycemia and dyslipidemia, as well as a decrease in the birth weight of their offspring [16–18]. Similarly, probiotics supplementation has been suggested to improve glycemic control via multiple mechanisms, such as reducing inflammation, enhancing the production of short-chain fatty acids (SCFAs), regulation of gut microbiota, improving insulin sensitivity, and preventing excessive weight gain [19, 20]. However, conflicting findings arise from previous studies examining the efficacy of probiotics in preventing GDM [21]. Two meta-analyses conducted previously did not find significant evidence to support the use of probiotics in reducing the risk of GDM [22, 23]. However, they included only five to six studies and significant heterogeneity, which were not explored due to the limited number of available studies, was observed in both [22, 23]. Additional randomized controlled trials have been published since [24–29]. Accordingly, the aim of our study was to perform an updated meta-analysis to comprehensively evaluate the influence of probiotics supplementation on the incidence of GDM in pregnant women.

## Materials and methods

This study is in accordance with the guidelines of Preferred reporting items for systematic reviews and meta-analyses (PRISMA) [30, 31] and the Cochrane Handbook [32].

### Study inclusion and exclusion criteria

The principle of PICOS, which is explained below, was utilized to determine the inclusion criteria for the meta-analysis.

P (participants): Women planning to conceive or at early pregnancy; I (intervention): Probiotics supplements during pregnancy, with no restrictions to the strains, timing, or dose of probiotics; C (control): Placebo or no additional treatment; O (outcomes): Reported the incidence of GDM during follow-up. The methods and criteria for the diagnosis of GDM were in accordance with those reported in the original studies. S (study design): Only RCTs with parallel groups that were published as complete articles in English or Chinese in peer-reviewed journals were deemed eligible for study design. Non-randomized studies, studies not including women planning to conceive or at early pregnancy, not with an intervention of probiotic supplementation, or not reporting the outcome of GDM incidence were excluded. In case studies with potentially overlapping patient populations were found, the meta-analysis included the one that had the larger sample size.

### Literature search strategy

To identify studies in Medline (PubMed), CENTER (Cochrane Library), Embase (Ovid), Web of Science, Wanfang, and China National Knowledge Infrastructure (CNKI), a search strategy was employed that encompassed the following criteria by a combination of the keywords: (1) “probiotic” OR “probiotics” OR “lactobacillus” OR “lactobacilli” OR “bifidobacteria” OR “bifidobacterium”; (2) “gestational diabetes mellitus” OR “GDM” OR [(“gestational” OR “pregnancy” OR “pregnant”) AND (“diabetes” OR “diabetic” OR “hyperglycemia”)]; and (3) “random” OR “randomized” OR “randomized” OR “randomly” OR “allocated” OR “control” OR “placebo.” Our focus was solely on research that involved human participants. In addition, we conducted a manual search for references to relevant reviews and primary articles. The most recent database search was conducted on December 21, 2023.

### Extraction of data and assessment of study quality

Two authors conducted separate searches in databases, gathered information, and assessed the quality. In case of any disagreements, the corresponding author was consulted to reach a consensus. For the study, various data were gathered including general details, characteristics of the study design, participant characteristics, age, BMI, proportions of women with primipara, use of lifestyle recommendations (diet and exercise), details of interventions (probiotics used, timing, and dose), regimens of controls, and criteria for the diagnosis of GDM. Cochrane’s Risk of Bias Tool [32] was used to evaluate the quality of RCTs included in this review. It assessed seven domains, including the generation of random sequence, concealment of allocations, blinding of participants and personnel, blinding of outcome evaluation, incomplete result data, and selective reporting of outcomes.

### Statistical analysis

The incidence of GDM, compared between women with probiotics supplementation and women in the control group, was summarized as risk ratio (RR) and corresponding 95% confidence interval (CI). The outcome data was extracted using the intention-to-treat principle. The Cochrane *Q* test was used to investigate the heterogeneity among the included studies [32]. Furthermore, the I^2^ statistic was calculated, where I^2^ > 50% suggested statistical heterogeneity [33]. To incorporate potential heterogeneity, a random-effect model was employed for pooling the data [32]. For outcomes of adequate datasets (10 or above), meta-regression and subgroup analyses according to study characteristics were performed to evaluate the source of heterogeneity. The meta-regression analysis tested the significance of the individual study characteristics’ influence on the results of the meta-analysis, with a *P* value < 0.05 indicating a significant modification effect. A positive coefficient demonstrated that the evaluated study characteristics are positively related to the OR of the results, while a negative coefficient demonstrated that the evaluated study characteristics are negatively related to the OR of the results. These characteristics included study country, mean age, BMI, timing, and dose of probiotics supplementation, and the risk of GDM of the studied females as reflected by the incidence of GDM in the control groups. Medians of continuous variables were selected as the cutoffs to define the subgroups. Publication bias was evaluated using Egger’s test for regression asymmetry and funnel plots [34]. A *P* value < 0.05 suggested a statistically significant distinction. The statistical analysis was conducted using RevMan (Version 5.1; Cochrane, Oxford, UK) and Stata (Version 12.0; Stata Corporation, USA) software.

## Results

### Literature search

**Figure 1.**
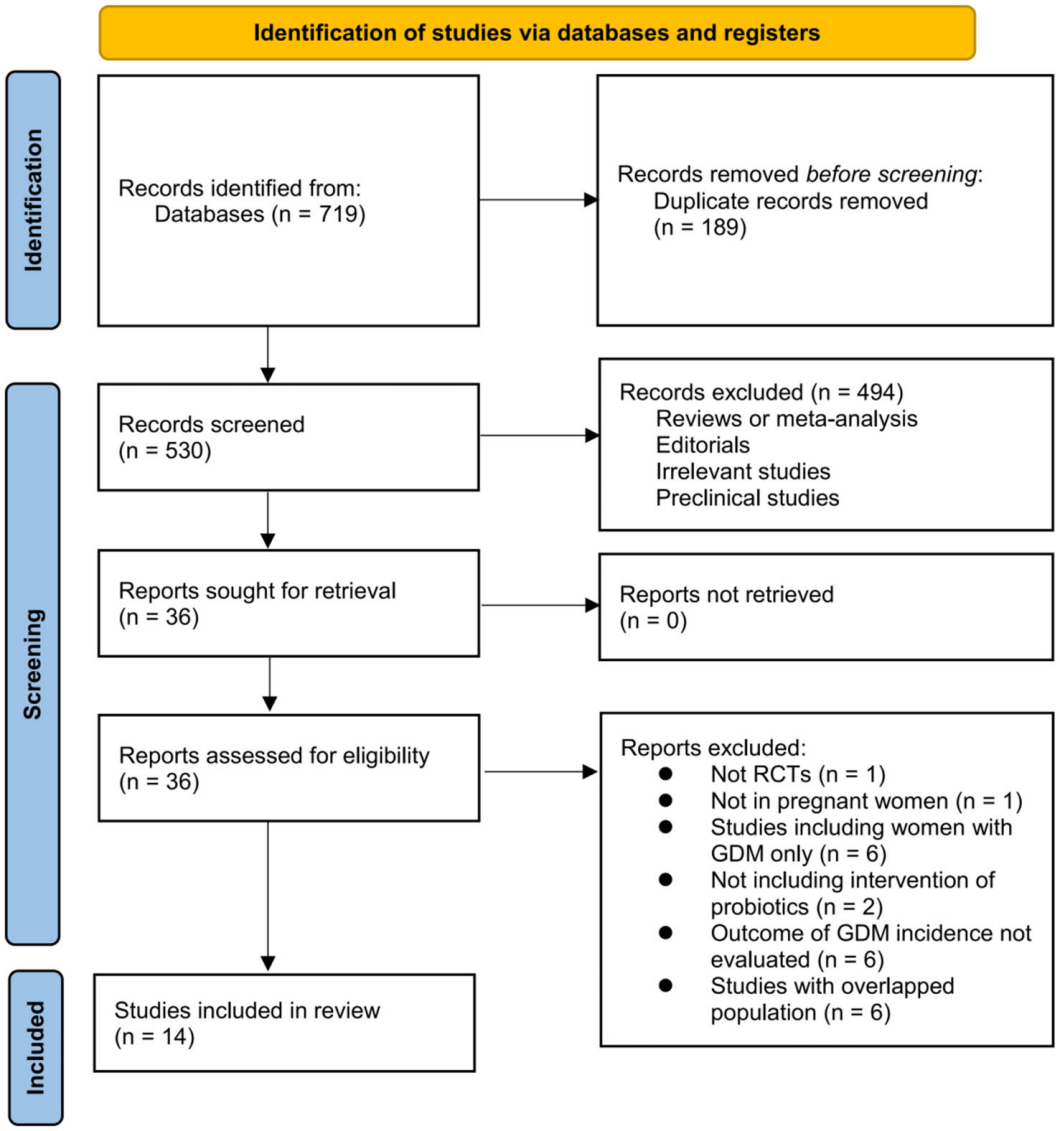
**Flowchart of the literature search.** GDM: Gestational diabetes mellitus.

The process of acquiring literature is illustrated in Figure 1. In summary, a total of 719 articles were obtained through database searches, with 530 remaining after removing duplicates. A total of 494 articles were subsequently excluded by screening via titles and abstracts, primarily because they were not relevant to the objective of the study. After reading the full text, an additional 22 articles out of the initial 36 were excluded due to the reasons outlined in Figure 1. At last, 14 RCTs [24–29, 35–42] were available for the subsequent meta-analysis.

### Study characteristics and data quality evaluation

Table 1 provides a summary of the studies included in the meta-analysis. In total, there were 14 RCTs involving 3527 females who were planning to conceive in the upcoming six months or at early pregnancy [24–29, 35–42]. These studies were published between 2010 and 2022 and carried out in Finland, Ireland, New Zealand, Australia, China, Denmark, Iran, the United Kingdom, Singapore, and Pakistan. The mean ages of the females were 27–34 years, and the mean BMI scores were 21–39 kg/m^2^. The proportions of females with primipara varied between 15.0%–63.5%. In four studies, dietary recommendation was also provided to females of the intervention and control groups [25, 35, 36, 41]. However, no evaluation has been performed regarding the diet or physical activities pre- and post-intervention among these studies. Multiple different strains were used for probiotics supplementation, such as *Lactobacillus rhamnosus*, *Lactobacillus acidophilus*, *Lactobacillus salivarius*, *Bifidobacterium lactis*, *Bifidobacterium longum*, *Bifidobacterium bifdum*, *Lactobacillus casei*, *Lactobacillus bulgaricus*, *Lactobacillus plantarum*, *Lactobacillus paracasei*, *Bifidobacterium breve*, *Bifidobacterium infantis*, and *Streptococcus thermophilus*, with *Lactobacillus rhamnosus* GG as the most commonly used probiotics strain. Most of the included studies used multiple strains as intervention except for three studies [36–38], in which single-strain probiotics were used. The timing for the starting of probiotics supplementation varied among the included studies, ranging from within the first trimester to the gestational age (GA) of 24 weeks. The total doses of probiotics were 1–50×10^9^ colony-forming units per day. As for the controls, placebo capsules were used in 12 studies [26–29, 35–42], while for the other two studies, no additional treatment was considered as controls [24, 25]. The incidence of GDM was diagnosed with the International Association of Diabetes in Pregnancy Study Group criteria [43] in all the studies using a “one-step” 2-h 75 g oral glucose tolerance test (OGTT) except for one study [36], in which GDM was diagnosed with the American College of Obstetricians and Gynecologists criteria using a “two-step” 3-h 100 g OGTT test [44]. Compliance data were reported in three studies, with similar mean adherence rates of 94.5% [37], 88.4% [40], and >90% [38] between females of the intervention and control groups, indicating good compliance. The incidence of adverse events was reported in two studies [26, 40]. Only mild discomfort related to the treatments was reported, which was similar in females in the intervention and control groups, with gastrointestinal symptoms being the most common symptoms.

**Table 1:** Characteristics of the included studies

**Study**	**Location**	**Design**	**Participants**	**Patient number**	**Mean age (years)**	**Mean BMI (kg/m^2^)**	**Primipara (%)**	**Lifestyle recommendations**	**Timing of intervention**	**Intervention**	**Total dose (10^9^ cfu/d)**	**Control**	**GDM diagnosis**
Luoto, 2010	Finland	R, DB, PC	Women at early pregnancy with no chronic metabolic diseases	152	29.9	23.6	57.9	Diet only	First trimester to delivery	*Lactobacillus rhamnosus* GG and *Bifidobacterium lactis* Bb12	20	Placebo capsule	IADPSG criteria
Lindsay, 2014	Ireland	R, DB, PC	Obese women at early pregnancy	138	31.2	33.6	44.9	Diet only	Second trimester (GA: 24 weeks) to delivery	*Lactobacillus salivarius* UCC118	1	Placebo capsule	ACOG criteria
Wickens, 2017	New Zealand	R, DB, PC	Pregnant women with a personal or partner history of atopic disease at early pregnancy	373	34	25.5	NR	NR	Second trimester (GA: 14∼16 weeks) to delivery	*Lactobacillus rhamnosus* HN001	6	Placebo capsule	IADPSG criteria
Okesene, 2019	New Zealand	R, DB, PC	Women at early pregnancy with BMI >30 kg/m^2^	230	28.7	38.6	31.7	No dietary recommendation	Second trimester (GA: 13∼17 weeks) to delivery	*Lactobacillus rhamnosus* GG and *Bifidobacterium lactis* Bb12	6.5	Placebo capsule	IADPSG criteria
Pellonpera, 2019	Finland	R, DB, PC	Women at early pregnancy with BMI >25 kg/m^2^	190	30.6	29.8	47.9	No dietary recommendation	Second trimester (GA: 18 weeks) to delivery	*Lactobacillus rhamnosus* HN001 and *Bifidobacterium animalis* ssp. lactis 420	20	Placebo capsule	IADPSG criteria
Callaway, 2019	Australia	R, DB, PC	Women at early pregnancy with BMI >25 kg/m^2^	411	31.5	31.8	38.7	NR	Second trimester (GA: 20 weeks) to delivery	*Lactobacillus rhamnosus* GG and *Bifidobacterium lactis* Bb12	2	Placebo capsule	IADPSG criteria
Wang, 2019	China	R, SB	Women at early pregnancy	400	27.2	21.5	NR	NR	Second trimester (GA: 16 weeks) to delivery	*Lactobacillus rhamnosus* GG and *Bifidobacterium lactis* Bb12	2	No additional treatment	IADPSG criteria
Halkjar, 2020	Denmark	R, DB, PC	Obese women at early pregnancy	49	30.7	31.9	NR	NR	Second trimester (GA: 14∼20 weeks) to delivery	*Streptococcus thermophilus* DSM 24,731, bifdobacteria and lactobacilli	45	Placebo capsule	IADPSG criteria
Cao, 2020	China	R, OL	Women at early pregnancy	100	33.8	23.6	50%	Diet only	Second trimester (GA: 13∼14 weeks) to delivery	*Lactobacillus rhamnosus* GG and HN001, *Limosilactobacillus tegmentum* CECT5716, *Bifidobacterium animalis* ssp. lactis HN019	15	No additional treatment	IADPSG criteria
Asgharian, 2020	Iran	R, DB, PC	Women at early pregnancy with BMI >25 kg/m^2^	128	29.5	29.8	15%	Diet only	Second trimester (GA: 24 weeks) to delivery	*Lactobacillus acidophilus* La5 and *Bifdobacterium lactis* Bb12	50	Placebo yoghurts	IADPSG criteria
Shahriari, 2021	Iran	R, DB, PC	Women at early pregnancy with high risk for GDM	507	32	30.2	NR	No dietary counseling	Second trimester (GA: 14 weeks) to delivery	*Lactobacillus acidophilus* LA1, *Bifdobacterium longum* sp54 cs, and *Bifdobacterium bifdum* sp9 cs	15	Placebo capsule	IADPSG criteria
Godfrey, 2021	United Kingdom, Singapore, and New Zealand	R, DB, PC	Women planning to conceive in upcoming 6 months	577	30.3	25.7	63.5	NR	Before pregnancy to delivery	*Lactobacillus rhamnosus* NCC 4007 and *Bifidobacterium animalis* subspecies lactis NCC 2818	2	Placebo capsule	IADPSG criteria
Baloch, 2022	Pakistan	R, DB, PC	Women at early pregnancy with high risk for GDM	160	30	26	NR	NR	Second trimester (GA: 13∼14 weeks) to delivery	*Streptococcus*, bifidobacteria and lactobacilli	5	Placebo capsule	IADPSG criteria
Liu, 2022	China	R, DB, PC	Women at early pregnancy	112	29.7	22.6	NR	NR	Second trimester (GA: 20 weeks) to delivery	*Streptococcus*, bifidobacteria and lactobacilli	12	Placebo capsule	IADPSG criteria

BMI: Body mass index; cfu: Colony-forming unit; GDM: Gestational diabetes mellitus; R: Randomized; DB: Double-blinded; PC: Placebo-controlled; OL: Open-label; SB: Single-blinded; GA: Gestational age; IADPSG: The International Association of Diabetes in Pregnancy Study Group; ACOG: The American College of Obstetricians and Gynecologists; NR: Not reported.

Table 2 provides a detailed analysis of the included RCTs using Cochrane’s Risk of Bias Tool. One of the included studies was open-label [25], another one was single-blinded [24], while the remaining 12 studies were double-blinded [26–29, 35–42]. The details of the random sequence generation were reported in nine studies [26, 27, 36–42], and seven studies reported the details of allocation concealment [27, 36, 38–42].

**Table 2:** Study quality evaluation via the Cochrane’s Risk of Bias Tool

**Study**	**Random sequence generation**	**Allocation concealment**	**Blinding of participants**	**Blinding of outcome assessment**	**Incomplete outcome data addressed**	**Selective reporting**	**Other sources of bias**
Luoto, 2010	Unclear	Unclear	Low risk	Low risk	Low risk	Low risk	Low risk
Lindsay, 2014	Low risk	Low risk	Low risk	Low risk	Low risk	Low risk	Low risk
Wickens, 2017	Low risk	Unclear	Low risk	Low risk	Low risk	Low risk	Low risk
Okesene, 2019	Low risk	Low risk	Low risk	Low risk	Low risk	Low risk	Low risk
Pellonpera, 2019	Low risk	Low risk	Low risk	Low risk	Low risk	Low risk	Low risk
Callaway, 2019	Low risk	Low risk	Low risk	Low risk	Low risk	Low risk	Low risk
Wang, 2019	Unclear	Unclear	High risk	Low risk	Low risk	Low risk	Low risk
Halkjar, 2020	Low risk	Low risk	Low risk	Low risk	Low risk	Low risk	Low risk
Cao, 2020	Unclear	Unclear	High risk	High risk	Low risk	Low risk	Low risk
Asgharian, 2020	Low risk	Low risk	Low risk	Low risk	Low risk	Low risk	Low risk
Shahriari, 2021	Low risk	Low risk	Low risk	Low risk	Low risk	Low risk	Low risk
Godfrey, 2021	Low risk	Unclear	Low risk	Low risk	Low risk	Low risk	Low risk
Baloch, 2022	Unclear	Unclear	Low risk	Low risk	Low risk	Low risk	Low risk
Liu, 2022	Unclear	Unclear	Low risk	Low risk	Low risk	Low risk	Low risk

### Influence of probiotics on the incidence of GDM

**Figure 2.**
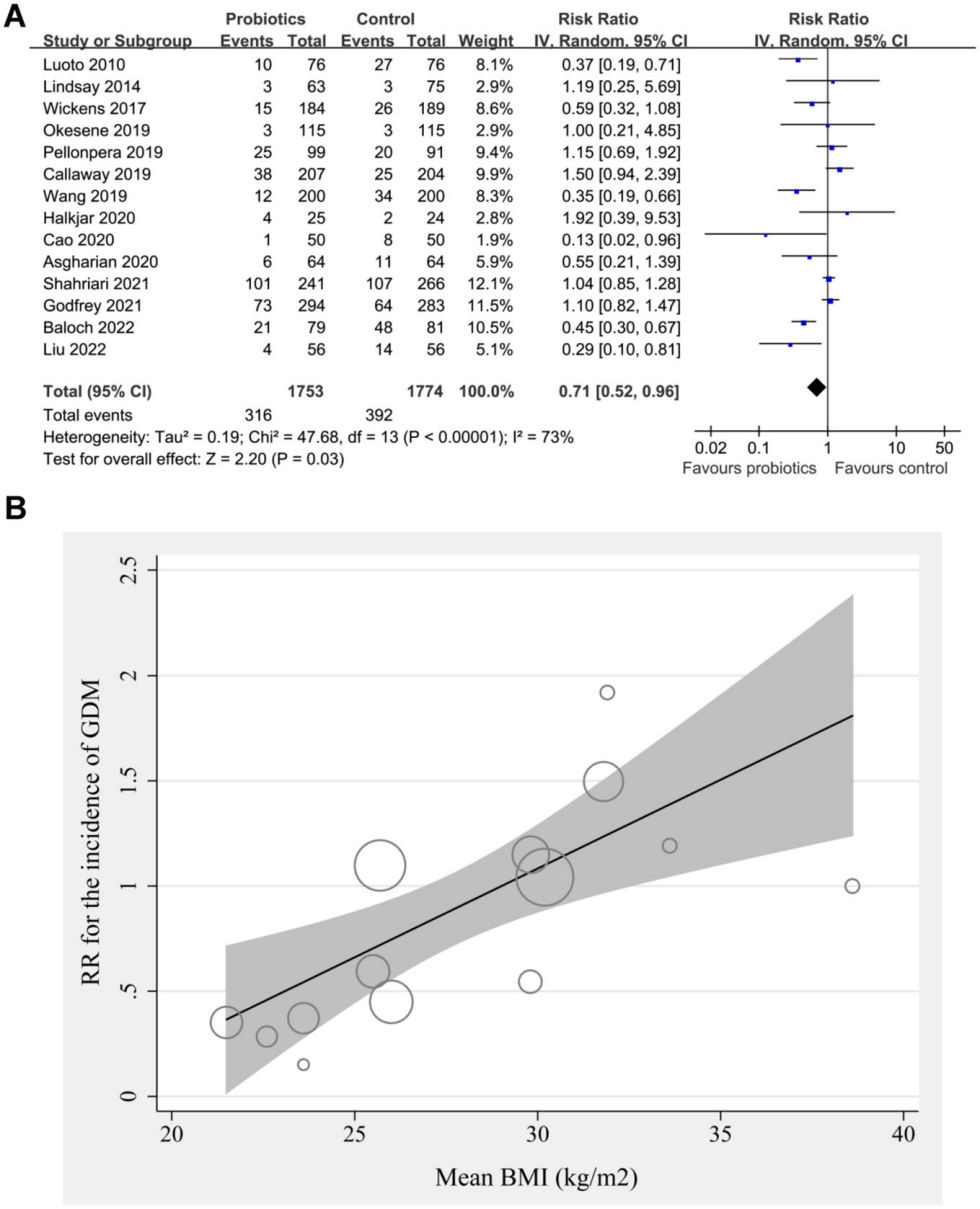
**Meta-analysis for the role of probiotics on the incidence of GDM in pregnant women.** (A) Forest plots for the overall meta-analysis of the influence of probiotics on the incidence of GDM; (B) Univariate regression analysis for the influence of BMI on the efficacy of probiotics for the prevention of GDM. RR: Risk ratio; CI: Confidence interval; GDM: Gestational diabetes mellitus; BMI: Body mass index; IV: Inverse variance.

Pooled results of 14 studies using random-effects models showed that probiotics significantly reduced the incidence of GDM as compared to control (RR: 0.71, 95% CI: 0.52–0.96, *P* ═ 0.03; Figure 2A) with significant heterogeneity (I^2^ ═ 73%). Sensitivity analysis by excluding the study with ACOG criteria [36] for the diagnosis of GDM retrieved similar results (RR: 0.70, 95% CI: 0.51–0.95, *P* ═ 0.02; I^2^ ═ 75%). In addition, a sensitivity analysis excluding the two studies with probiotics started at the 24 weeks of GA [36, 41] also showed similar results (RR: 0.71, 95% CI: 0.51–0.98, *P* ═ 0.04; I^2^ ═ 76%).

The meta-regression showed that the females’ mean BMI was positively associated with the RR for the effect of probiotics on GDM (coefficient ═ 0.084, *P* ═ 0.01; Figure 2B and Table 3), which largely explained the source of between-study heterogeneity (residual I^2^ ═ 10.5%). Other variables such as sample size, mean age, probiotics dose, median GA for starting probiotics, or incidence of GDM in control groups were not suggested to be significant modifiers for the effect of probiotics on GDM, according to the results of the meta-regression analyses (*P* all > 0.05, Table 3).

**Table 3:** Results of univariate meta-regression analysis

**Variables**	**RR for the incidence of GDM**			
	**Coefficient**	**95% CI**	***P* values**	***I*^2^ residual**
Sample size	0.0010	0.0004–0.0024	0.15	35.2%
Mean age (years)	0.082	−0.081–0.244	0.30	45.6%
BMI (kg/m^2^)	0.084	0.025–0.144	0.01	10.5%
Dose of probiotics (10^9^ cfu/d)	−0.00057	−0.02342–0.02227	0.96	53.0%
Median GA for starting probiotics	0.0048	−0.0398–0.0495	0.82	53.1%
Incidence of GDM in control group (%)	−0.010	−0.027–0.007	0.23	46.3%

RR: Risk ratio; GDM: Gestational diabetes mellitus; CI: Confidence interval; BMI: Body mass index; cfu: Colony-forming unit; GA: Gestational age.

**Figure 3.**
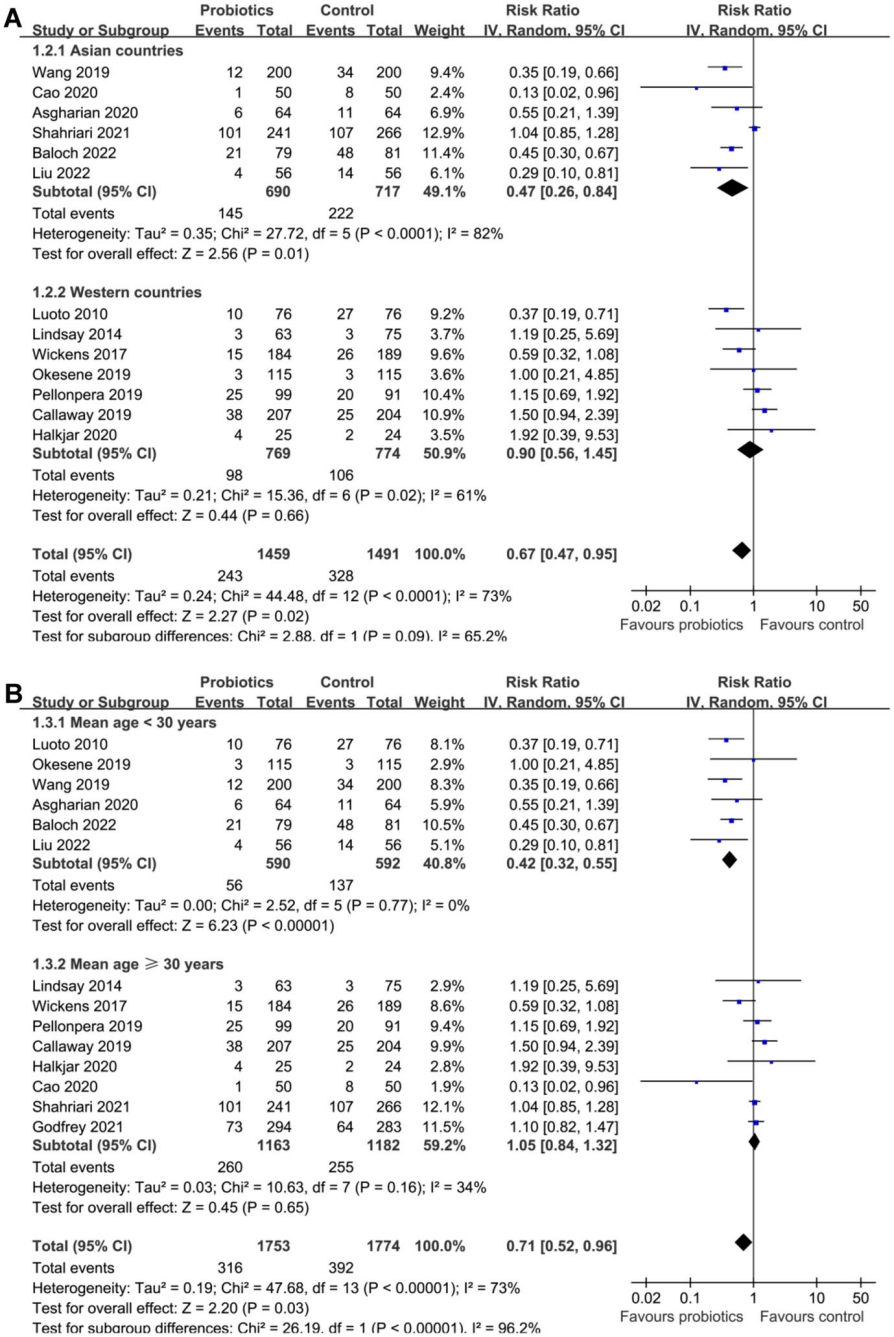
**Forest plots for the subgroup analyses of the efficacy of probiotics for the prevention of GDM**. (A) Subgroup analysis based on country and (B) based on mean ages. GDM: Gestational diabetes mellitus; CI: Confidence interval; IV: Inverse variance.

**Figure 4.**
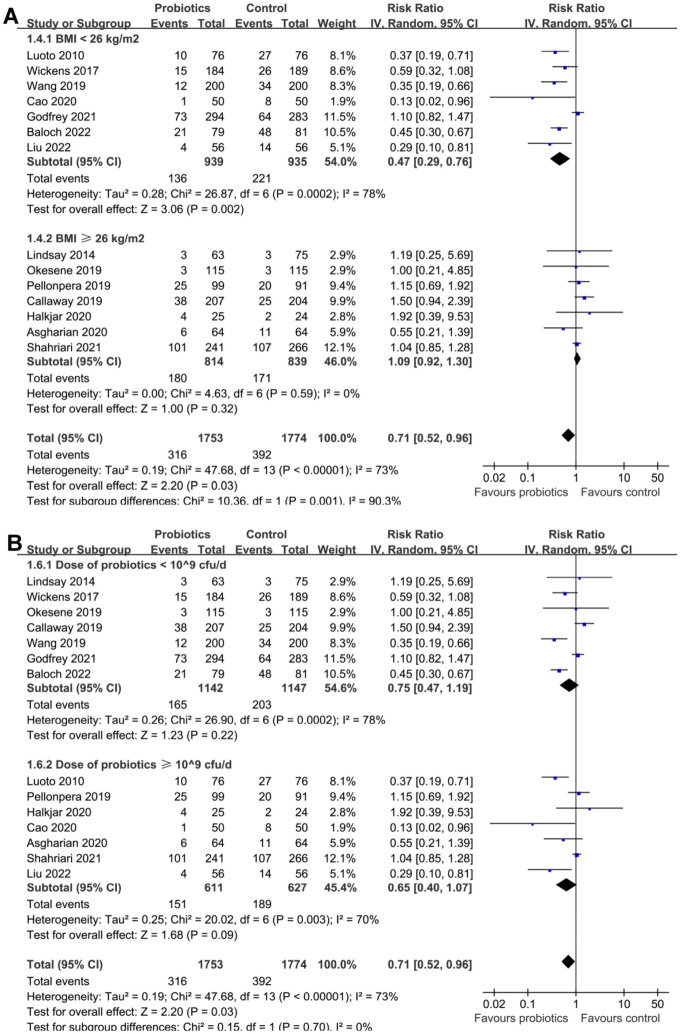
**Forest plots for the subgroup analyses of the efficacy of probiotics for the prevention of GDM.** (A) Subgroup analysis based the mean BMI of women and (B) subgroup analysis based on the probiotics dose. GDM: Gestational diabetes mellitus; BMI: Body mass index; CI: Confidence interval; cfu: Colony-forming unit; IV: Inverse variance.

**Figure 5.**
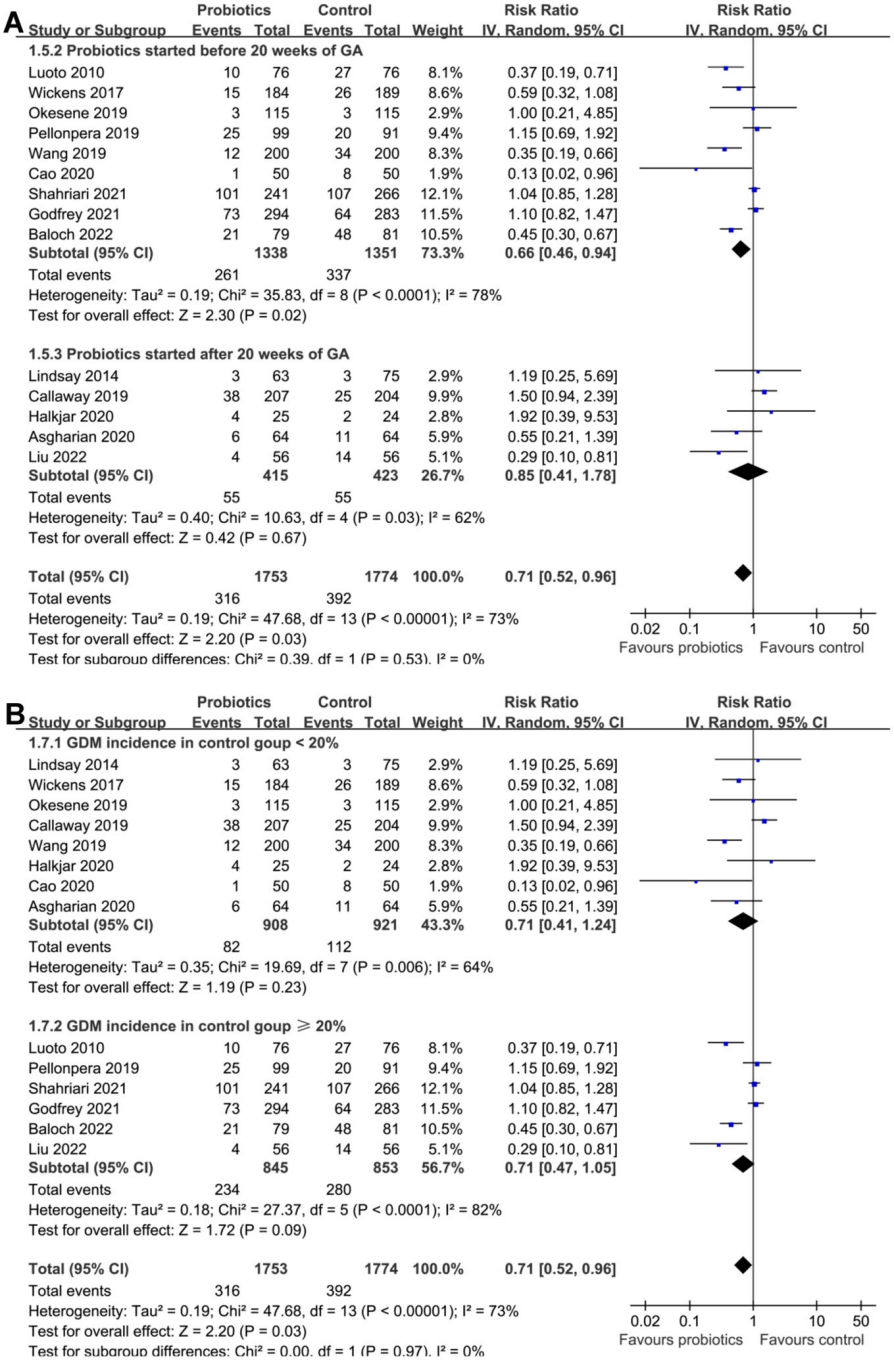
**Forest plots for the subgroup analyses of the efficacy of probiotics for the prevention of GDM.** (A) Subgroup analysis based on the timing of probiotics supplementation and (B) based on the risk of GDM of the included women as reflected by the incidence of GDM in control groups. GDM: Gestational diabetes mellitus; CI: Confidence interval; GA: Gestational age; IV: Inverse variance.

Subsequent subgroup analyses according to the study country did not significantly affect the results (*P* for subgroup difference ═ 0.09; Figure 3A). However, the results of subgroup analyses indicated that the preventative efficacy of probiotics on GDM was remarkable in females < 30 years, but not in those ≥30 years (RR: 0.42 vs 1.05, *P* for subgroup difference < 0.001; Figure 3B). In addition, it was also indicated that probiotics significantly reduced the risk of GDM in females with BMI < 26 kg/m^2^, but not in those with BMI ≥ 26 kg/m^2^ (*P* for subgroup difference ═ 0.001; Figure 4A). Subgroup analyses did not support that other study characteristics could significantly influence the effect of probiotics supplementation on the risk of GDM, such as probiotics dose (*P* for subgroup difference ═ 0.70; Figure 4B), timing of probiotic supplementation (*P* for subgroup difference ═ 0.53; Figure 5A), or risk of GDM as reflected by the incidence of GDM in controls (*P* for subgroup difference ═ 0.97; Figure 5B).

### Publication bias

**Figure 6.**
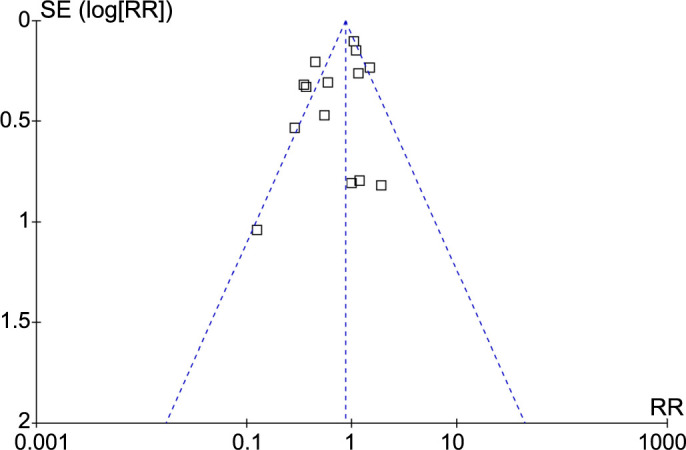
**Funnel plots evaluating the publication bias of the meta-analysis for the role of probiotics on the incidence of GDM in pregnant women.** GDM: Gestational diabetes mellitus; RR: Risk ratio.

The funnel plots for the meta-analysis of the probiotics’ influence on the incidence of GDM in pregnant women are shown in Figure 6. The funnel plots are symmetrical on visual inspection, suggesting the low risk of publication bias. The results of Egger’s regression test also suggested a low risk of publication bias (*P* ═ 0.39).

## Discussion

In our study, by pooling the results of 14 RCTs, we found that probiotics supplementation during pregnancy could significantly reduce the incidence of GDM. Interestingly, subsequent meta-regression and subgroup analyses suggested that the BMI of the pregnant females may significantly modify the effect of probiotics on GDM, which largely explained the source of heterogeneity. Specifically, probiotics significantly reduced the risk of GDM in women with BMI < 26 kg/m^2^, but not in those with BMI ≥ 26 kg/m^2^. In addition, the preventative efficacy of probiotics on GDM was remarkable in women < 30 years, but not in those ≥ 30 years. Taken together, the results of this meta-analysis indicate that probiotics may be effective in reducing the risk of GDM, particularly for females with lower BMI and younger age.

Although several meta-analyses have been published on the topic of the influence of probiotics supplementation on the risk of GDM [22, 23], this current updated meta-analysis has several methodological strengths compared to the previous ones. First, in this meta-analysis, we performed an extensive literature search in six commonly used electronic databases, and retrieved eligible RCTs which investigated the efficacy of probiotics for the prevention of GDM. As a result, 14 studies involving 3527 pregnant females were included. The overall sample size of the meta-analysis is much larger than that of the previous ones [22, 23]. In addition, multiple meta-regression and subgroup analyses were performed to identify the study characteristics’ influences on the outcome and to determine the source of heterogeneity.

We found that the BMI of the females was positively associated with the RR of the probiotic’s effect on GDM, and probiotics significantly reduced the risk of GDM in females with BMI < 26 kg/m^2^, but not in those with BMI ≥ 26 kg/m^2^. Similarly, the preventative efficacy of probiotics on GDM was remarkable in women < 30 years, but not in those ≥ 30 years. The mechanisms underlying these findings remain to be determined. Interestingly, it has been confirmed that advanced maternal age [45] and obesity [46] are established risk factors for GDM. Therefore, the findings of this study may suggest that probiotics supplementation is effective in reducing the risk of GDM in low-risk women, but not in high-risk women. Physiologically, the mechanisms underlying the effects of probiotics supplementation during pregnancy are to attenuate the gut dysbiosis related to pregnancy, a potential pathway linked to the pathogenesis of GDM [47]. For females with high risk for GDM, multiple mechanisms may be involved in the pathogenesis of GDM besides dysregulation of intestinal microbiota, such as islet beta-cell dysfunction, insulin resistance, neurohormonal dysregulation, oxidative stress, and inflammation [48], and probiotics supplementation may become less effective. In addition, a previous study suggested that multi-strain probiotics are beneficial for improved metabolic and inflammatory outcomes in post-GDM women by modulating gut dysbiosis, which highlighted the necessity for a comprehensive strategy for postpartum treatment that includes probiotics to protect post-GDM women from developing glucose intolerance [49]. Accordingly, females with advanced age and obesity may respond poorly to probiotics because they have lesser gut microbial diversity. Moreover, a recent meta-analysis indicated that probiotics may have positive effects on metabolic, inflammation, oxidative stress, and neonatal outcomes in females with GDM. Additionally, diet and pre-intervention washout may modify the effects of probiotics [50]. These factors may also confound the efficacy of probiotics in pregnant females with high risk for GDM. These hypotheses should be validated in future studies.

This meta-analysis has limitations. First, the species/strains of probiotics varied among the included studies, which may also lead to heterogeneity. Future studies should be performed to determine the optimal species/strains for the prevention of GDM. Second, although our meta-regression and subgroup analyses did not show that differences in dose or timing for starting probiotics supplementation may modify the effect of probiotics for the prevention of GDM, the optimal dose and timing for starting probiotics remain to be clarified in this clinical setting. Third, the incidence of GDM could be significantly affected by dietary habits and physical activities [51], two key factors that may modify the potential preventative efficacy of probiotics on GDM. However, these two factors were rarely reported or controlled among the included studies. In addition, most of the studies did not evaluate the baseline gut microbial diversity and did not observe the effect of probiotics on gut microbial diversity after intervention. Finally, this meta-analysis was based on study-level data rather than individual patient-level data. Accordingly, results of the meta-regression and subgroup analyses should be interpreted with caution. Large-scale RCTs are still needed to validate these findings.

## Conclusion

Taken together, probiotics supplementation may be effective in reducing the risk of GDM, particularly for females with lower BMI and young age. Although the optimal species/strains, dose, and starting timing of probiotics supplementation remain to be determined, these findings support the potential use of probiotics supplementation as an effective strategy to reduce the incidence of GDM in pregnant females. Further research is needed to evaluate the influence of probiotic supplementation on the risk of GDM in high-risk females, such as those with advanced age and obesity, especially high-quality RCTs.

## Data Availability

All data generated during the study are presented in the manuscript.
